# Genetic risk for schizophrenia and autism, social impairment and developmental pathways to psychosis

**DOI:** 10.1038/s41398-018-0229-0

**Published:** 2018-09-26

**Authors:** Eva Velthorst, Sean Froudist-Walsh, Eli Stahl, Douglas Ruderfer, Ilyan Ivanov, Joseph Buxbaum, iPSYCH-Broad ASD Group, the IMAGEN consortium, Anders D. Børglum, Jakob Grove, Manuel Mattheisen, Thomas Werge, Preben Bo Mortensen, Marianne Giørtz Pedersen, Carsten Bøcker Pedersen, Ole Mors, Merete Nordentoft, David M. Hougaard, Jonas Bybjerg-Grauholm, Marie Bækvad-Hansen, Christine Søholm Hansen, Mark J. Daly, Benjamin M. Neale, Elise B Robinson, Felecia Cerrato, Ashley Dumont, Jacqueline Goldstein, Christine Stevens, Raymond Walters, Claire Churchhouse, Stephan Ripke, Joanna Martin, Tobias Banaschewski, Arun L. W. Bokde, Uli Bromberg Dipl-Psych, Christian Büchel, Erin Burke Quinlan, Sylvane Desrivières, Herta Flor, Vincent Frouin, Hugh Garavan, Penny Gowland, Andreas Heinz, Bernd Ittermann, Marie-Laure Paillère Martinot, Eric Artiges, Frauke Nees, Dimitri Papadopoulos Orfanos, Tomáš Paus, Luise Poustka, Sarah Hohmann, Juliane H. Fröhner, Michael N. Smolka, Henrik Walter, Robert Whelan, Gunter Schumann, Abraham Reichenberg

**Affiliations:** 1 Department of Psychiatry and Seaver Autism Center for Research and Treatment, Icahn School of Medicine at Mount Sinai, New York, USA; 2 Center for Neural Science, New York University, New York, NY 10003 USA; 3 Institute for Genomics and Multiscale Biology, Icahn School of Medicine at Mount Sinai, New York, NY 10029 USA; 4 Vanderbilt Genetics Institute, Nashville, USA; 5 Department of Child and Adolescent Psychiatry and Psychotherapy, Central Institute of Mental Health, Medical Faculty Mannheim, Heidelberg University, Square J5, 68159 Mannheim, Germany; 6 Discipline of Psychiatry, School of Medicine and Trinity College Institute of Neuroscience, Trinity College Dublin, Dublin, Ireland; 7 University Medical Centre Hamburg-Eppendorf, House W34, 3.OG, Martinistr. 52, 20246 Hamburg, Germany; 8 Medical Research Council - Social, Genetic and Developmental Psychiatry Centre, Institute of Psychiatry, Psychology & Neuroscience, King’s College London, London, United Kingdom; 9 Department of Cognitive and Clinical Neuroscience, Central Institute of Mental Health, Medical Faculty Mannheim, Heidelberg University, Square J5, Mannheim, Germany; 10 Department of Psychology, School of Social Sciences, University of Mannheim, 68131 Mannheim, Germany; 11 NeuroSpin, CEA, Université Paris-Saclay, F-91191 Gif-sur-Yvette, France; 12 Departments of Psychiatry and Psychology, University of Vermont, 05405 Burlington, Vermont USA; 13 Sir Peter Mansfield Imaging Centre School of Physics and Astronomy, University of Nottingham, University Park, Nottingham, United Kingdom; 14 Department of Psychiatry and Psychotherapy, Campus Charité Mitte, Charité, Universitätsmedizin Berlin, Charitéplatz 1, Berlin, Germany; 15 Physikalisch-Technische Bundesanstalt (PTB), Braunschweig and Berlin, Braunschweig, Germany; 16 Institut National de la Santé et de la Recherche Médicale, INSERM Unit 1000 “Neuroimaging & Psychiatry”, University Paris Sud – Paris Saclay, University Paris Descartes; and AP-HP, Department of Adolescent Psychopathology and Medicine, Maison de Solenn, Cochin Hospital, Paris, France; 17 INSERM, Research Unit 1000 “Neuroimaging and Psychiatry”, Service Hospitalier Frederic Joliot, Orsay, France; 18 University Paris Sud – Paris Saclay, Orsay, France; 19 University Paris Descartes, Paris, France; 20 GH Nord Essonne, Psychiatry Department 91G16, Orsay Hospital, Orsay, France; 21 Rotman Research Institute, Baycrest and Departments of Psychology and Psychiatry, University of Toronto, Toronto, Ontario M6A 2E1 Canada; 22 Department of Child and Adolescent Psychiatry and Psychotherapy, University Medical Centre Göttingen, von-Siebold-Str. 5, 37075 Göttingen, Germany; 23 Clinic for Child and Adolescent Psychiatry, Medical University of Vienna, Währinger Gürtel 18-20, 1090 Vienna, Austria; 24 Department of Psychiatry and Neuroimaging Center, Technische Universität Dresden, Dresden, Germany; 25 School of Psychology and Global Brain Health Institute, Trinity College Dublin, Dublin, Ireland; 26 iPSYCH, The Lundbeck Foundation Initiative for Integrative Psychiatric Research, Aarhus, Denmark; 27 iSEQ, Center for Integrative Sequencing, Aarhus University, Aarhus, Denmark; 28 Department of Biomedicine - Human Genetics, Aarhus University, Aarhus, Denmark; 29 Bioinformatics Research Centre, Aarhus University, Aarhus, Denmark; 30 Center for Neonatal Screening, Department for Congenital Disorders, Statens Serum Institut, Copenhagen, Denmark; 31 Psychosis Research Unit, Aarhus University Hospital, Risskov, Denmark; 32 Institute of Biological Psychiatry, MHC Sct. Hans, Mental Health Services Copenhagen, Roskilde, Denmark; 33 Department of Clinical Medicine, University of Copenhagen, Copenhagen, Denmark; 34 National Centre for Register-Based Research, Aarhus University, Aarhus, Denmark; 35 Centre for Integrated Register-based Research, Aarhus University, Aarhus, Denmark; 36 Mental Health Services in the Capital Region of Denmark, Mental Health Center Copenhagen, University of Copenhagen, Copenhagen, Denmark; 37 Analytic and Translational Genetics Unit, Massachusetts General Hospital, Boston, USA; 38 Stanley Center for Psychiatric Research Broad Institute of MIT and Harvard, Cambridge, USA; 39 Department of Epidemiology, Harvard Chan School of Public Health, Boston, Massachusetts USA; 40 Department of Psychiatry, Charite Universitatsmedizin Berlin Campus Benjamin Franklin, Berlin, Germany; 41 MRC Centre for Neuropsychiatric Genetics and Genomics: Cardiff, Cardiff, United Kingdom; 42 Department of Medical Epidemiology and Biostatistics, Karolinska Institutet, Stockholm, Sweden

**Keywords:** Genomics, Neuroscience, Schizophrenia, Autism spectrum disorders

## Abstract

While psychotic experiences (PEs) are assumed to represent psychosis liability, general population studies have not been able to establish significant associations between polygenic risk scores (PRS) and PEs. Previous work suggests that PEs may only represent significant risk when accompanied by social impairment. Leveraging data from the large longitudinal IMAGEN cohort, including 2096 14-year old adolescents that were followed-up to age 18, we tested whether the association between polygenic risk and PEs is mediated by (increasing) impairments in social functioning and social cognitive processes. Using structural equation modeling (SEM) for the subset of participants (*n* = 643) with complete baseline and follow-up data, we examined pathways to PEs. We found that high polygenic risk for schizophrenia (*p* = 0.014), reduced brain activity to emotional stimuli (*p* = 0.009) and social impairments in late adolescence (*p* < 0.001; controlling for functioning in early adolescence) each independently contributed to the severity of PEs at age 18. The pathway between polygenic risk for autism spectrum disorder and PEs was mediated by social impairments in late adolescence (indirect pathway; *p* = 0.025). These findings point to multiple direct and indirect pathways to PEs, suggesting that different processes are in play, depending on genetic loading, and environment. Our results suggest that treatments targeting prevention of social impairment may be particularly promising for individuals at genetic risk for autism in order to minimize risk for psychosis.

## Introduction

An increasing number of studies on the etiology of schizophrenia and related disorders focus on psychotic experiences (PEs) as early and potentially powerful markers of illness liability^1^. PEs are mild expressions of psychotic symptoms that are present in about 10%^2^ of the general population, and are known precursors of severe psychotic and non-psychotic disorders^3,4^.

However, the etiology of PEs is unknown. Most previous general population studies examining genome-wide association study (GWAS)-based polygenic risk scores (PRS) have not been able to find evidence for a significant association between increased genetic vulnerability to psychiatric illness and PEs in the general population^1,5–7^. While it has been argued that this lack of evidence may indicate that, within the healthy population, risk manifests through symptoms other than psychotic experiences^5,7^, it may also indicate that, in and of itself, experiencing subclinical psychotic phenomena does not represent risk. The latter premise is supported by recent studies in high-risk samples, suggesting that PEs increase the chance of developing a clinically significant psychiatric disorder only when accompanied by increasing and persistent social impairments, irrespective of the baseline severity of PEs^8,9^. Severe mental disorders are often preceded by impairments in interpersonal contact^10,11^, and the association between subclinical psychotic symptoms and impairments in interpersonal social functioning is well established^12,13^. This latter association may also help explain why, within the general population, apart from those with genetic risk for schizophrenia (SCZ), individuals with genetic risk for autism spectrum disorder (ASD) are at a considerably higher risk for developing PEs later in life^14^. Social communicative deficits that contribute to impairments in interpersonal functioning characterize ASD^15^, and it seems probable that ASD is a vulnerability factor for the development of PEs^14,16^, potentially due to an association between social impairment and psychosis.

In this study, we tested whether the association between genetic risk for psychiatric illness and PEs is mediated by (increasing) impairments in social functioning, using a Structural Equation (pathway) Model that minimized assumptions about interactions between variables. As associations may differ for those with an increased genetic vulnerability to SCZ and increased genetic vulnerability to ASD, we explored both genetic risk variants. We expected that an increased genetic risk for SCZ and ASD would be associated with an increased risk of PEs at age 18, and that this association would be at least partly mediated by (increasing) social impairments for both risk variants.

Our data came from the IMAGEN study^17^; a longitudinal study that has the unique advantage of having followed-up individuals from the ages 14 to 18, a critical time for the development of peer relationships as well as the development of psychotic symptoms^18–20^. With this dataset, we also had the opportunity to link measures of reported difficulties in social functioning with fMRI measures of brain activity while participants viewed salient social and emotional stimuli^21–23^. Brain activation in response to emotional stimuli has been associated with the severity of social impairment in ASD^24,25^ and SCZ populations^26^.

## Materials and methods

### Participants and procedures

The IMAGEN study is a multi-site multi-national longitudinal research project^17^. This collaboration between eight European sites across the United Kingdom, Ireland, France and Germany includes 2462 14-year-old adolescents and their parents. The study protocol was approved by the KCL (King’s College London) College Research Ethics Committee CREC/06/07-71 and by local ethics research committees at each site. Parents and adolescents gave written consent and verbal assent, respectively. Biosamples, brain imaging, clinical characteristics, and functioning data were collected at baseline. Behavioral assessments were repeated at 2 and 4 years after completion the baseline. A detailed description of recruitment and research procedures has been published elsewhere^17^.

A total of 2096 IMAGEN participants (1066 females and 1030 males, *M* age at baseline = 14.45 years (range 12.89–16.44; SD = 0.41) had complete socio-demographic data and no formal DSM-IV social disorder diagnosis (see Supplementary material for dropout analyses). Table 1a lists the demographic details of the study sample.

**Table 1:** Characteristics of 2096 volunteers participating in the IMAGEN study

	1a. Total study sample (*n* = 2096)	1b. Subsample with complete data for SEM analyses (*n* = 642)	Statistics
Gender ratio male/female; *n* (%)	1030 (49.14)/1066 (50.86)	301 (46.88)/341 (53.12)	*χ*^2^(1) = 1.9, *p* = 0.17
Baseline age; *M* (SD)	14.46 (0.41)	14.44 (0.43)	*t* = −1.33, *p* = 0.18
*Participation rate per center; n (*%*)*			
London	262 (12.76)	103 (16.04)	*χ*^2^(7) = 26.8, *p* < 0.001^a^
Nottingham	313 (15.24)	76 (11.84)	
Dublin	205 (9.98)	60 (9.35)	
Berlin	262 (12.76)	63 (9.81)	
Hamburg	263 (12.80)	123 (19.16)	
Mannheim	237 (11.54)	67 (10.44)	
Paris	254 (12.37)	78 (12.15)	
Dresden	258 (12.56)	72 (11.21)	
IQ estimate raw score; *M* (SD)	38.14 (5.06)	39.53 (4.29)	*t* = 6.30, *p* < 0.001
*Clinical characteristics*			
Social functioning baseline; *M* (SD)	6.38 (1.37)	6.41 (1.39)	*t* = 0.66, *p* = 0.51
Social functioning year 3; *M* (SD)	6.24 (1.29)	6.32 (1.25)	*t* = 2.06, *p* = 0.04
CAPE psychotic experiences; *M* (SD)	11.29 (6.29)	11.30 (5.80)	*t* = 0.04, *p* = 0.97
CAPE depression score; *M* (SD)	4.27 (2.75)	4.27 (2.61)	*t* = -0.07, *p* = 0.94
CAPE negative symptom score; *M* (SD)	4.51 (3.59)	4.53 (3.43)	*t* = 0.13, *p* = 0.89

Range social functioning = 1–8 (higher scores indicate better functioning)

*Estimate IQ raw score* raw score (WISC Vocabulary + WISC Matrix Reasoning), *CAPE* community assessment of psychic experiences (range positive: 0–43, depression: 0–17, negative: 0–30)

^a^Follow-up *χ*^2^ analyses indicate that participants from London (*χ*^2^(1) = 25.3, *p* < 0.001) and Hamburg (*χ*^2^(1) = 53.1, *p* < 0.001) were significantly overrepresented in the SEM analyses

### Measures of clinical characteristics and functioning

Social functioning was determined using the strength and difficulties questionnaire (SDQ;^27^ Combining self and parental reports, this scale provides a dimensional assessment of emotional problems (anxiety-depression), peer problems, conduct problems, hyperkinetic symptoms, and pro-social behavior. In the current study, we analyzed responses from the peer problem domain, as this subscale aligns most closely with the social impairments commonly reported in schizophrenia and related psychotic disorders^20^, but is also known to be compromised in individuals with an autism spectrum disorder^15^. Questions in the peer problem domain include “I am usually on my own”, “I have one good friend or more”, “Other people my age generally like me”, “I get on better with adults than with people my age”, and “Other children or young people pick on me”. Because, the latter item taps into bullying, a known risk factor for the development of PEs^28^, and therefore a potential confounder, this item was removed from analyses. All items were rated on a three-point scale, with scores ranging from ‘Not True’ to ‘Always True’. Two items were recoded so that for all items higher scores indicated better functioning.

IQ was estimated by averaging sum scores of the WISC-IV subscales Matrix Reasoning (fluid IQ marker) and Vocabulary (crystalized IQ marker)^29^.

PEs were assessed using the community assessment of psychic experiences (CAPE) which evaluates subclinical psychotic experiences in the affective and non-affective domains (http://www.cape42.homestead.com/). The CAPE is a self-report questionnaire based on the Peters et al. Delusions Inventory^30^ but with the addition of questions on hallucinatory experiences. We used the sum score on frequency of positive symptoms.

### Measures of genetic vulnerability

IMAGEN genetic data, extracted from whole-blood samples (~10 mL) using Illumina 610Quad v1 chip, were accessed through the IMAGEN consortium^17^. GWAS was performed at Centre National de Genotype (Evry France, Head M Lathrop). In order to access high-quality data combined across IMAGEN waves, QC + genotyped SNPs (*n* = 477,245, list provided by IMAGEN) were extracted from downloaded imputed, hard-called data and filtered for 0% missing data. Further quality control (QC) included exclusion of SNPs with Hardy Weinberg equilibrium HWE *P* < 10–4 and SNPs with minor allele frequency (MAF <2%). After quality control, 1834 cases were included in our sample, totaling 463,940 SNPs available for PRS analysis. Ten MDS (multidimensional scaling) components were also downloaded from IMAGEN, and were used as covariates in the analyses. MDS is a singular-value decomposition number based on an individual-pairwise identity by relation matrix, and is essentially equivalent to PCA but implemented in plink. This method is standard to generate genotype ancestry covariates. All genetic data processing and analyses were performed using plink^31^. Details on (the processing of) the ASD^32^ and SCZ^33^ summary statistics are presented in Table 2.

**Table 2:** Processing of the ASD and SCZ GWAS summary statistics

GWAS summary statistics
The ASD summary statistics are based on results from a meta-analysis of 5305 trios of European ancestry from the PGC autism sample and 18,381 ASD cases and 27,969 controls of European ancestry from the iPSYCH autism sample. A description of the PGC sample is available on the PGC web site: https://www.med.unc.edu/pgc/files/resultfiles/PGCASDEuro_Mar2015.readme.pdf and in ref. ^32^. Briefly, five cohorts provided genotypes (*n* denote the number of trios for which genotypes were available): The Geschwind Autism Center of Excellence (ACE; *n* = 391), the Autism Genome Project (AGP; *n* = 2272)^49^, the Autism Genetic Resource Exchange (AGRE; *n* = 974)^50^, the NIMH Repository (https://www.nimhgenetics.org/available_data/autism/), the Montreal/Boston Collection (MONBOS; *n* = 1396)^51^, and the Simons Simplex Collection (SSC; *n* = 2231)^52^.
The iPSYCH ASD sample is based on the population-based case-cohort sample iPSYCH2012^53^, which is extracted from the birth cohorts consisting of all children born in Denmark between 1 May 1981 and 31 December 2005. Eligible were singletons born to a known mother and resident in Denmark on their 1st birthday. Cases in iPSYCH2012^54^ were defined from the Danish Psychiatric Central Research Register^55^ with diagnosis date no later than 2012, and the controls constitute a random sample from the set of eligible children. Cases in the iPSYCH ASD sample are defined as subjects in iPSYCH2012^53^ having an ASD diagnosis (ICD codes F84.0, F84.1, F84.5, F84.8, or F84.9) given no later than 2013, and controls did not have an ASD diagnosis by 2013. The samples were linked using the unique personal identification number to the Danish Newborn Screening Biobank. Genotypes are available on 14,970 cases and 26,125 controls.
Data processing and QC were conducted according to the standards employed by the PGC Statistical Analysis Group and carried out using their pipeline Ricopili^56^. To minimize potential batch effects the data were processed separately in the 23 genotyping batches in the case of iPSYCH and for each cohort in the PGC sample. Phasing was achieved using SHAPEIT^57^ and imputation done by IMPUTE2^58,59^ with haplotypes from the 1000 Genomes Project, phase 3 (1kGP3)^60^ as reference. Trio samples were imputed as a case-pseudo-controls design. PCA was carried out using smartPCA^61,62^ on independent SNPs and unrelated samples (plink identity by state *π* ^ < 0.2). In iPSYCH, a subsample was taken with all parents and grandparents known to have been born in Denmark (*n* = 31,500), and with European ancestry defined by the first 3 principal components (PCs). In the PGC sample a European ancestry subsample was taken using a first 3 PCs weighted Euclidian distance from CEU + TSI HapMap populations (±10 standard deviations). A second PCA provided covariates for the association analyses. Association analyses were logistic regression in plink 1.9 using the imputed dosages for each iPSYCH batch and PGC cohort. The results were subsequently meta analyzed using METAL^63^ (July 2010 version) employing an inverse variance weighted fixed effect model^64^. We filtered the summary statistics allowing only markers with an imputation info score ≥0.7, maf ≥0.01 and effective sample size 4 × Nca × Nco/(Nca + Nco), where Nca and Nco are case and control Ns, respectively, at least 70% of the maximum.
GWAS summary statistics for SCZ (35k cases, 43k controls^33^) were downloaded from the Psychiatric Genomics Consortium (http://www.med.unc.edu/pgc/results-and-downloads), and for Autism (18,381 cases and 27,969 controls, all European decent; unpublished) were acquired through collaboration. We QC filtered the 1000 Genomes phase 1 imputed GWAS summary data (Autism: sample size Neff > 0.7 total, imputation INFO score ≥ 0.3; SCZ: Ndatasets ≥ 35 out of 52 for SCZ, INFO > 0.3).
ASD and SCZ summary statistics were aligned to our IMAGEN SNP list (461,568 SNPs remaining in SCZ; 455,972 in ASD), and then LD pruned based on *P*-value using plink2 --clump with an LD r2 threshold of 0.25 (final SNPs remaining 133,194 for SCZ and 129,973 for Autism). We performed polygenic scoring with GWAS *P*-value thresholds 10^−^^4^, 10^−2^, 0.1, and 0.5 in the IMAGEN data.

### Neuroimaging measure of social cognitive processing

The imaging task of interest was the Faces task^17^, measuring emotional reactivity to social stimuli^34^. Participants were asked to passively view short videos of either faces that always started from a neutral position, and then either (1) morphed to an angry expression, or (2) displayed a neutral movement, or (3) displayed a non-biological control stimulus that consisted of contracting or expanding concentric circles of contrasts roughly matching that of the faces stimuli^34^.

Functional magnetic resonance imaging (fMRI) was performed on 3T scanners from a range of manufacturers (Siemens, Philips, General Electric, Bruker) across the eight IMAGEN assessment sites.

A total of 1060 volumes per subject were obtained, each containing 40 2.4 mm slices (with a 1 mm gap), with a repetition time of 2.2 s and an echo time of 30 ms. Data preprocessing was performed centrally at the Neurospin centre using the SPM12 software. Time-series data were corrected for slice-timing effects and motion, and then nonlinearly warped to MNI space (using a custom EPI template), and Gaussian smoothed at 5 mm full width at half maximum.

Individual contrast maps for the ‘angry minus control’ contrast from the Faces task were created in SPM using the general linear model with the AR noise model. Twenty-one additional regressors of no interest were added to the design matrix, comprising 12 motion regressors, 3 white matter regressors and 6 nuisance variables for the ventricles.

We then created a mask of regions thought to be involved in social processing. First, we used the automatic meta-analysis tool ‘NeuroSynth’ to create a reverse-inference brain map for the term ‘social’ on the basis of 1000 fMRI studies of social functioning^35^. This map was already registered to the MNI152 (2 mm) space. In order to co-register the map to the IMAGEN functional data, we created a warp from the MNI brain to the IMAGEN EPI200 brain using the ANTs normalization software with a mutual information cost function^36^. This warp was then applied to the reverse inference map obtained from NeuroSynth using nearest neighbor interpolation. For more specific information about data acquisition and fMRI data preprocessing, we would like to refer to refs ^17,37^.

### Statistical analyses

Demographic characteristics of the baseline and available follow-up samples were compared using regression analyses and *χ*^2^-tests.

### Preparatory analyses

Initial analyses were conducted to explore what specific PRS and fMRI data to include in the final integrative structural equation modeling (SEM) pathway analysis.

#### Polygenic risk scores

First, we examined which polygenic risk thresholds to consider in subsequent analyses by exploring the association (*R*^2^) between SCZ polygenic risk (PRS) *P*-value thresholds and PEs at age 18, and between ASD polygenic risk (ASD-PRS) and social functioning at the same age using linear regression analyses. To examine the unique variance (*R*^2^) explained by the polygenic risk scores, age, sex, research center, and ancestry (MDS) coordinates were included as covariates and the PRS residuals were retained for regression analyses.

#### fMRI processing

In order to parsimoniously capture each participant’s fMRI response to viewing angry faces, we calculated the principal component projection of fMRI BOLD activity in response to viewing angry faces (vs baseline)^17^ in the social cognition network. The social cognition network was identified as the ten regions with the highest *z*-score (and cluster extent) from an automatic meta-analysis of 1000 fMRI studies of social processing^35^, as in refs ^38,39^.

### Structural equation modeling (SEM)

Pathway analyses were constructed to model the relationship between genetic risk, social impairment at age 14 and 18, social cognitive (brain) processing and the eventual emergence of PEs. Sex, research center and IQ were added as potential contributing factors to the model. SEM provides estimates, or path coefficients, that indicate the direction and significance of the association between constructs, as well as several fit indices evaluating the fit of the proposed model.

For acceptable model fit, the established and widely used cutoff values for SEM as described by Hu and Bentler were used^36^. Following these rules, *χ*^2^ (chi-square) should be non-significant (meaning that model is congruent with observed data), root mean square error of approximation (RMSEA) should be lower than 0.05, and the comparative fit index (CFI) should be higher than 0.90. Under population error, the RMSEA value is reported along with lower and upper bounds of its 90% confidence interval. The standardized root mean square residual (SRMR) is the standardized difference between the observed and predicted correlation, and considered acceptable with values at 0.08 or less^40^. The comparative fit index (CFI) considers the number of paths in the model and is considered good at 0.93 or above. Finally, we considered the bayesian information criterion (BIC), where no absolute value is indicative of good model fit, but lower values of BIC represent a better fit. Both direct and indirect effects (path estimates) were examined.

In our final pathway model, we began with a fully saturated model, including IQ, sex, research center, ASD-PRS, SCZ-PRS, fMRI data on social cognitive processing, social impairment at age 14 and 18, and PEs to examine their interrelationship, and removed non-significant pathways to produce models with the optimal balance of explanatory power and parsimony. We used a maximum-likelihood approach, only including those with all data points available. For sensitivity purposes, analyses were repeated using a maximum-likelihood analysis with missing values, and potential mediating pathways were confirmed with a Sobel-Goodman test (SGMEDIATION).

All main analyses were conducted in STATA 14.2(Statacorps).

## Results

### PRS thresholds

As shown in Table 3, the predictive value was highest for a SCZ-PRS threshold of *p* ≤ 1e−2 (*R*^2^ = 0.0063, *t* = 2.55, *p* = 0.010), and similar for an ASD-PRS threshold of *p* ≤ 0.5 & *p* ≤ 1 (*R*^2^ = 0.0044, *t* = −2.18, *p* = 0.030), and the SCZ-PRS threshold of *p* ≤ 1e−2 & ASD-PRS threshold of *p* ≤ 0.5 were used in subsequent analyses. In these analyses, MDS coordinates were included as covariates in the prediction of PRS and the residuals were retained.

**Table 3:** Predictive value of SCZ polygenic risk scores (PRS) on psychotic experiences, and of ASD polygenic risk scores (PRS) on social functioning scores

Predictive value of SCZ polygenic risk scores (PRS) on psychotic experiences					
*p*-value threshold	*R*^2^ variance explained	*t*	*p*	Coefficient	95% CI
*p* ≤ 1e−4	0.0027	1.69	0.093	491.86	−82.97–1066.70
*p* ≤ 1e−2	0.0063	2.55	0.010*	2796.82	677.28–4916.36
*p* ≤ 0.1	0.0043	2.10	0.036*	5592.94	372.23–10,813.64
*p* ≤ 0.5	0.0033	1.84	0.067	10,644.87	−734.84–22,024.58
*p* ≤ 1	0.0032	1.82	0.070	14,820.22	−1193.02–30,833.46

Predictive value of ASD polygenic risk scores (PRS) on social functioning scores					
*p*-value threshold	R^2^ variance explained	*t*	*p*	Coefficient	95% CI
*p* ≤ 1e–4	0.0030	−1.81	0.071	−34.98	−72.96–2.99
*p* ≤ 1e–2	0.0009	−.99	0.324	−138.85	−414.76–137.05
*p* ≤ 0.1	0.0031	−1.83	0.086	−765.77	−1588.26–56.72
*p* ≤ 0.5	0.0044	−2.18	0.030*	−2123.26	−4037.85 to −208.68
*p* ≤ 1	0.0044	−2.18	0.029*	−3267.24	−6203.56 to −330.92

Results based on varying SNP *P*-value inclusion thresholds. Psychotic experiences were measured with the comprehensive assessment of psychic experiences sum score, and social impairments were measured with the peer problems sum score of the strength and difficulties questionnaire. To examine the unique variance (*R*^2^) explained by the polygenic risk scores, age, sex, research centre, and ancestry (MDS) coordinates were included as covariates and the PRS residuals were retained for regression analyses

*SCZ* schizophrenia, *ASD* autism spectrum disorder

*p < 0.05

### Brain areas involved in social cognitive processing

**Fig. 1:**
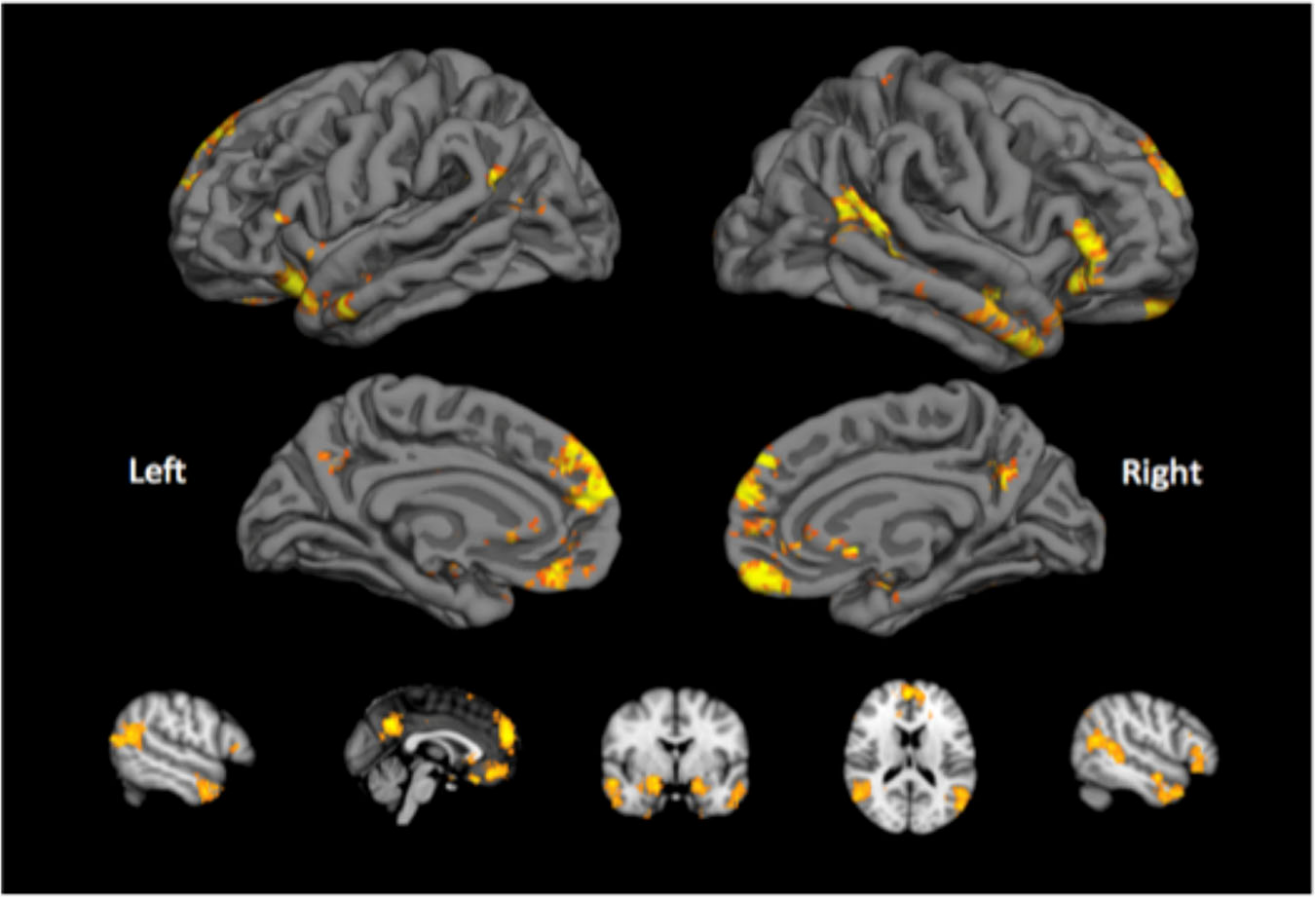
Brain regions involved in processing social (cognitive) information. An automatic meta-analysis using NeuroSynth identified a network ofregions involved in social processing. This network overlaps with the Default Mode Network, and includedthe dorsomedial and ventromedial prefrontal cortex, precuneus, temporal pole, and amygdala. Activity inthese regions during processing of faces was examined, and used as a predictor of psychotic experiences

With the meta-analytic tool we identified bilateral clusters in the dorsomedial and ventromedial prefrontal cortex, posterior cingulate, temporal pole, and amygdala that were significantly associated with social processing. These ten clusters with the highest *z*-score (also largest in size) were used for further analysis (see Fig. 1 and supplementary material). A principal component analysis including beta-values of the ‘angry faces minus control’ contrasts in these areas identified a single component that explained over 50% of the variance within the ‘social brain’ network. This principal component assigned similar weights to activity in all ten regions, and subject loadings along this component were entered as one of the variables in the pathway models.

### Pathway model

For the main SEM model, we limited our analyses to the 642 participants (341 females) of whom a complete baseline and follow-up dataset was available (see Table 1b for a description of the sub sample used in SEM analyses). Model statistics of the fitted path models are presented in Table 4. For a visual display of all models, and correlation matrix of all variables included see Supplementary Material.

**Table 4:** Model fit statistics

Model	*χ*^2^, *p*	DF	RMSEA	CFI	SRMR	BIC
Fully connected model	26.17, *p* < 0.001	7	0.065 (0.040–0.093)	0.879	0.030	22,063
Without PRSscz&asd → IQ	3.53, *p* = 0.62	5	0 (0–0.046)	1	0.012	22,054
Without PRSscz&asd → SF baseline	3.67, *p* = 0.82	7	0 (0–0.030)	1	0.012	22,041
Without PRSscz → SF follow-up	4.17, *p* = 0.84	8	0 (0–0.027)	1	0.012	22,035
Without PRSscz&asd → fMRI social	4.71, *p* = 0.91	10	0 (0–0.017)	1	0.013	22,022
Without PRSasd → PEs	4.90, *p* = 0.94	11	0 (0–0.010)	1	0.013	22,016
Without PEs → SF follow-up	5.87, *p* = 0.92	12	0 (0–0.014)	1	0.014	22,011
Without IQ → SF follow-up	6.09, *p* = 0.94	13	0 (0–0.007)	1	0.014	22,004
Without sex → SF baseline&fMRIsoc	6.91, *p* = 0.96	15	0 (0–0)	1	0.015	21,992
Without IQ → SF baseline	6.73, *p* = 0.82	11	0 (0–0.026)	1	0.014	22,018
Without IQ → PEs	8.55, *p* = 0.74	12	0 (0–0.029)	1	0.016	22,013
Without IQ → fMRI social	6.38, *p* = 0.78	10	0 (0–0.029)	1	0.016	18,299
Without sex → SF follow-up	10.73, *p* = 0.47	11	0 (0–0.041)	1	0.021	18,297

*χ*^2^ difference tests showed that models did not significantly worsen when removing connections up to model (M), which had significantly worse fit indices than model (L) (*p* < 0.05)

*PRS* polygenic risk score, *scz* schizophrenia, *asd* autism spectrum disorder, *SF* social functioning, *PEs* psychotic experiences, *fMRIsoc* fMRI social cognition

**Fig. 2:**
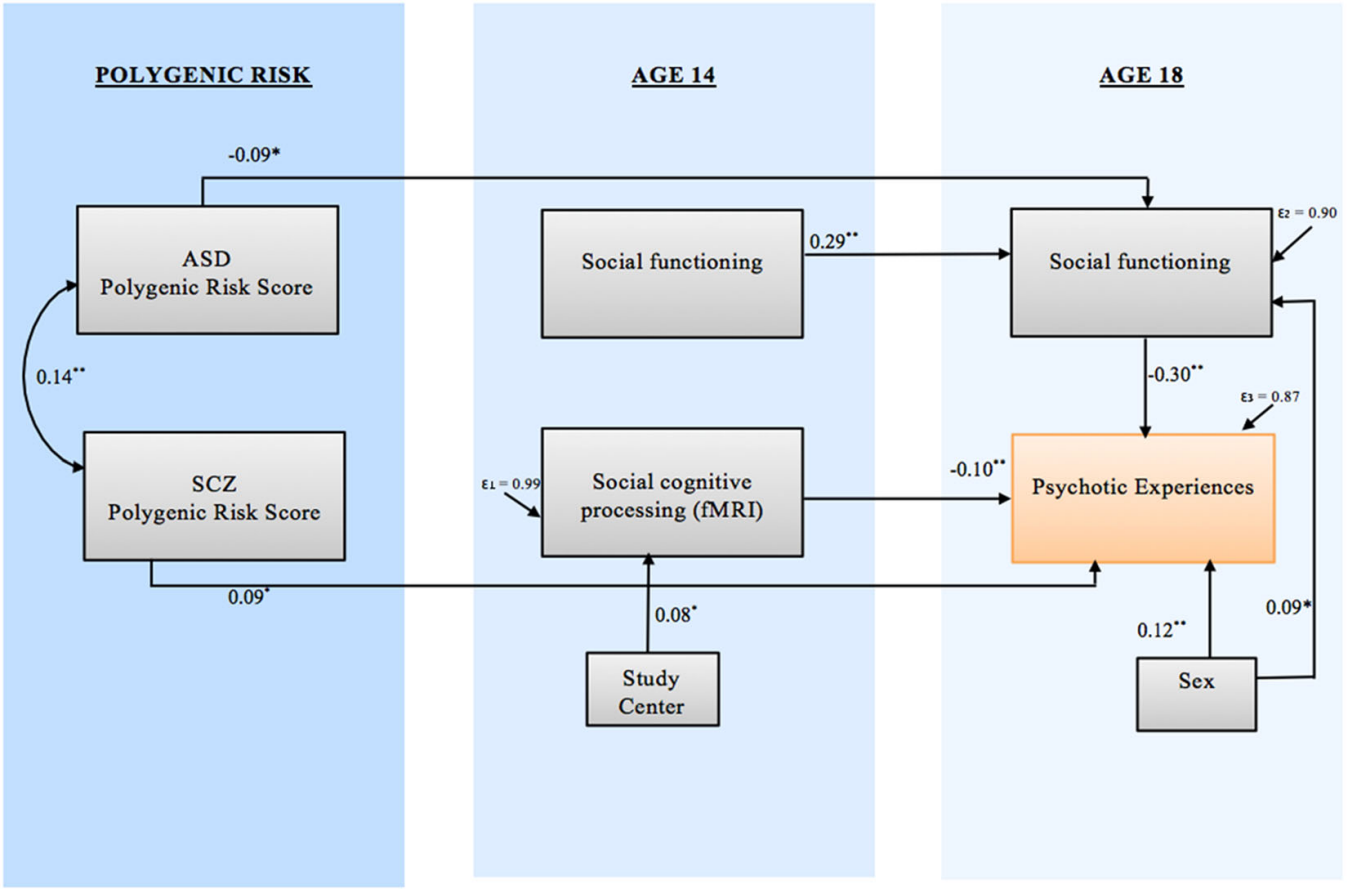
Final path model. Associations between the observed variables are represented by straight arrow lines. The double-headed arrow represents covariance between the two polygenic risk scores. Information about all coefficients and co-variances can be found in the supplementary figure. **p < 0.01, *p < 0.05. ASD autism spectrum disorder, SCZ schizophrenia

Model L (visually presented in Fig. 2) was the best fitting model. The overall model (*χ*^2^(10) = 6.38, *p* = 0.78; RMSEA = 0.000; 90% CI = 0.000–0.029, SRMR = 0.016; CFI = 1.00) accounted for 12% of the variance in PEs at age 18. The model suggested a direct path leading from increased genetic polygenic risk for SCZ to a higher number of reported PEs (standardized coefficient = 0.09, SE = 0.04, *Z* = 2.47, *p* = 0.014). Another direct pathway led from brain altered brain activation in response to ‘social emotional’ stimuli to PEs (standardized coefficient = −0.10, *p* = 0.009), indicating that a lower brain response in the ‘social brain’ network was associated with more PEs. The model also revealed that the association between ASD-PRS and a higher number of PEs at age 18 was mediated by peer problems that had evolved during the period between ages 14 and 18 (stand. indirect coefficient = 0.03, *p* = 0.025). In this model, social functioning at the age of 14 did not have a direct independent effect on the development of PEs by age 18. In addition, in our best fitting model, IQ did not significantly contribute to the explanation of PEs.

While a model allowing an additional direct causal pathway from PEs to social functioning at age 18 resulted in almost equally acceptable fit (*χ*^2^(10) = 5.28, *p* = 0.81; RMSEA = 0.000; 90% CI = 0.000–0.028, SRMR = 0.015; CFI = 1.000), this direct pathway was not statistically significant (stand. coefficient = 0.13, *p* = 0.316).

### Sensitivity analyses

To confirm that using a sample with complete data did not bias our results analyses were repeated, including data of all participants. An SEM analysis using maximum-likelihood with missing values approach yielded largely similar results (see Supplementary results).

In addition, a Sobel-Goodman mediation test (including 1003 individuals with available PRS, social functioning and PE data) was employed to examine whether the mediating effect of social functioning in the pathway from ASD-PRS to PEs held in a simplified model. Also in these exploratory analyses, the effect of ASD-PRS on PEs was significantly mediated by social functioning at follow-up (proportional mediation effect = 23.3%, *p* = 0.03), while for SCZ-PRS no mediation effect of social functioning was apparent (proportional mediation effect = 12.8%, *p* = 0.30).

## Discussion

The present study provides initial evidence for multiple pathways leading to the development of PEs. In our sample, severity of subclinical PEs at age 18 was independently predicted by (1) greater polygenic risk for schizophrenia, (2) poorer social functioning, and (3) brain activation in response to emotional stimuli. In our study, all regions within the ‘social cognition network’ contributed to the prediction of psychotic experiences to a similar degree, suggesting that disruption to the overall social cognition network may be critical to the development of psychotic experiences.

In contrast to previous studies^1,5–7^, our results indicate a small but statistically significant direct association between PRS for schizophrenia and PEs, although they also suggest that this is likely not the ‘single pathway’ to PEs. There is wide heterogeneity in the presentation of PEs, and different types of PEs (e.g., hallucinatory, delusions) may have different etiologies^41^. There is also heterogeneity in presentation of the clinical disorders which may help explain why interventions targeting the behavior and those aimed at altering neurobiological function have only been effective in treating subsections of patient populations^42^.

We also found an indirect pathway suggesting that the association between genetic risk for ASD and psychotic experiences may be mediated by social impairment that appears between age 14 and 18. This finding is consistent with recent molecular-genetic and population-based studies that indicate genetic and familial overlap between ASD and SCZ^43–45^, and with studies finding increased psychosis rates in individuals with ASD^14,46^.

Collectively, our findings strengthen the idea that social impairment is a strong predictor of PEs largely independent of genetic risk or irregular neural emotion-processing. The stronger association between PEs and social impairment at age 18 than at age 14 suggests that the period in between may be particularly important for the development of social impairments that constitute risk for psychosis.

Although the association between social impairment and psychotic manifestations is well-established, the direction of this relationship is unclear^13^. While the pathway from social impairment to PEs showed significantly better fit than a pathway from PEs to social impairment, causality should be interpreted cautiously. It is not inconceivable that the association between social impairment and PEs involves a dynamic feedback loop; while initial impairments in interpersonal contact may contribute to the development of certain delusions, these PEs may subsequently reinforce social impairment, further reinforcing PEs. To better understand the potential mechanisms underlying the association between social impairment and PEs, further longitudinal studies are required.

## Limitations

The results of this study should be interpreted in light of some key limitations. First, there was a relatively high level of missing data (>30%) in our key variables of interest. Although findings were comparable when we estimated missing values in SEM analyses (supplementary results) and by repeating the mediating pathways using a more conservative mediation model, we did not want to over-interpret our findings by imputing such a large proportion of biological and clinical data. Therefore, our main analyses included only those individuals with all data points available, reducing the sample size considerably.

Second, while the overall fit of our SEM model was good, we cannot rule out that an alternative model may have fitted our data equally well.

Third, when testing the association between brain activation and both genetic vulnerability and social impairments, we examined only one aspect of the brain network related to social cognitive processing which may have underestimated the association. Investigations of the relationship between structural or functional connectivity in this network, or of brain activation on other tasks in relation to genetic vulnerability and social impairments could offer complementary information.

Fourth, measures of social functioning were obtained using a relatively short assessment tool, and it may be that other aspects of social functioning not captured with this tool have different associations with the development of psychotic experiences. Nonetheless, the instrument used here captured the key problems known to be prominent in adolescents with both an autism spectrum disorder and those vulnerable to psychosis.

Fifth, because PEs were only assessed at age 18, we have no data on their onset. Future studies to the potential mechanisms underlying the association between social impairment and PEs should include PE measures collected on multiple time points and more detailed social functioning measures.

Sixth, the reported associations between PRS and outcome are small, accounting for only around 0.5% of variance in psychotic experiences and functioning. It is therefore important to note that these findings are solely of theoretical interest, as they point to different potential pathways to the development of psychosis.

Finally, it is important to note that ASD and SCZ polygenic risk scores are not unitary constructs. To disentangle the differential association between ASD and SCZ polygenic risk scores and psychotic experiences, it will be crucial to dissect their genetic correlation and non-overlapping risk.

Overall, our results provide new insight about potential etiological pathways to psychotic experiences. We found that poor social functioning at age 18 was related to both increased polygenic risk for ASD and more psychotic experiences at that age indicating a cross-diagnostic role of social impairment in psychiatric illness. Social contact is thought to be an important source of support in times of stress^47^, and the association between persistent social disengagement and stress-regulation (a known risk factor of psychotic symptoms^48^) warrants further study. Our results suggest that treatments targeting social impairment may be particularly promising for individuals at genetic risk for autism in order to minimize risk for psychosis.

It is also critical that future clinical studies use multi-faceted measures to determine whether our findings can be generalized to individuals after first hospitalization, and thus, to determine whether the pathways as described in our study actually lead to a clinical psychotic disorder. If multiple pathways can be detected, these findings urge caution in the exclusive assignment of therapy to psychosis, since it is probable that different processes are in play depending on genetic loading, or environment.

## Electronic supplementary material

Supplement 1. Measurement of Axis I diagnosis

Supplement 2. fMRI data preparation and initial processing

Supplement 3. Dropout analyses

Supplement 4. Different SEM models tested

Supplement 5. Sensitivity analyses

Supplement 6. Correlation matrix of variables included in the pathway models
