# Supplementary material for: Genetic risk for schizophrenia and autism, social impairment and developmental pathways to psychosis
Source: Transl Psychiatry. 2018 Sep 26;8:204. doi: 10.1038/s41398-018-0229-0 (PMC6158250; doi:10.1038/s41398-018-0229-0)
Supplement: Supplementary file 1 — Supplement 1. Measurement of Axis I diagnosis [file 41398_2018_229_MOESM1_ESM.docx]

Supplement 1. Measurement of Axis I diagnosis

Participants with any history of an axis 1 disorder related to social functioning (e.g. social phobia, social anxiety disorder) were excluded (n=28). Adolescent psychiatric symptoms and their impact were assessed with the Development and Well-Being Assessment (DAWBA), a self-administered diagnostic questionnaire consisting of open and closed questions (http://www. dawba.info). The DAWBA is designed to maintain consistency across multiple cultural and language groups, as clinical raters share a common training and participates in regular cross-language training and consensus meetings. The DAWBA generates probabilities of having DSM-IV-TR diagnoses(24), which we used to define the categorical psychiatric diagnosis (e.g. autism spectrum disorder, posttraumatic stress disorder and depression. For this study, everyone with a likelihood of >70 % of having a psychiatric disorder associated with social impairment was omitted from analyses
