# Supplementary material for: Genetic risk for schizophrenia and autism, social impairment and developmental pathways to psychosis
Source: Transl Psychiatry. 2018 Sep 26;8:204. doi: 10.1038/s41398-018-0229-0 (PMC6158250; doi:10.1038/s41398-018-0229-0)
Supplement: Supplementary file 2 — Supplement 2. fMRI data preparation and initial processing [file 41398_2018_229_MOESM2_ESM.docx]

**Supplement 2. fMRI data preparation and initial processing**

An automatic meta-analysis of fMRI activations related to the term ‘social’ revealed a network of regions including midline regions of the prefrontal and parietal lobe, and bilateral anterior temporal lobe (temporal pole), amygdala, posterior superior temporal lobe (temporo-parietal junction) and inferior frontal gyrus. These top 10 clusters were extracted from the reverse-inference map, and were in order of descending size:

Supplementary table 1. Top 10 cluster rank of brain regions involved in social processing

| Cluster rank | Region | Cluster size mm^3^ | max Z-score | peak x (mm) | peak y (mm) | peak z (mm) |
| --- | --- | --- | --- | --- | --- | --- |
| 1 | dmPFC | 13336 | 9.63 | 2 | 58 | 20 |
| 2 | rTPJ | 7656 | 7.28 | 50 | -44 | 10 |
| 3 | vmPFC | 6184 | 7.47 | 0 | 26 | -24 |
| 4 | rTPole | 5944 | 7.77 | 54 | 12 | -34 |
| 5 | lTPole/IFG | 5848 | 7.43 | -36 | 24 | -18 |
| 6 | lTPJ | 5200 | 8.36 | -58 | -60 | 20 |
| 7 | PCC/precuneus | 4528 | 9.26 | -2 | -56 | 36 |
| 8 | rAmygdala | 3336 | 7.82 | 18 | -2 | -14 |
| 9 | rIFG | 3120 | 8.13 | 46 | 26 | -12 |
| 10 | lAmygdala | 1984 | 7.03 | -20 | -6 | -18 |

*Details of the ten largest clusters obtained from the automated meta-analysis of fMRI studies of ‘social’ functioning. Cluster co-ordinates are listed in MNI space. Abbreviations: PFC = prefrontal cortex. r = right. l = left. dm = dorsomedial. vm = ventromedial. TPJ = temporo-parietal junction. TPole = temporal pole. PCC = posterior cingulate cortex. IFG = inferior frontal gyrus.*
