# Supplementary material for: Genetic risk for schizophrenia and autism, social impairment and developmental pathways to psychosis
Source: Transl Psychiatry. 2018 Sep 26;8:204. doi: 10.1038/s41398-018-0229-0 (PMC6158250; doi:10.1038/s41398-018-0229-0)
Supplement: Supplementary file 3 — Supplement 3. Dropout analyses [file 41398_2018_229_MOESM3_ESM.docx]

Supplement 3. Dropout analyses

The amounts of complete and reliable data differed per study area, and dropout analyses were therefore done separately for each outcome of interest. Participants omitted from GWAS analyses did not differ from the included sample in terms of age, gender, and negative symptomatology. The excluded sample did present with more psychotic experiences and depression symptoms at age 18 (coef = -1.15; t=-2.26, p= 0.024, 95%CI= -2.14 – -0.15; coef = -0.54, t=-2.44, p=0.015, 95%CI = -.98 – -.107 respectively), and had a significantly lower IQ estimate (coef= 1.76, t=5.81, p<0.001, 95%CI= 1.17 - 2.35). The individuals dropped from fMRI analyses were comparable to the rest of the sample in their gender, reported psychotic experiences, negative symptoms and in their reported depression, but were slightly younger (in days; coef= 56.06, t=2.62, p=0.009, 95%CI= 14.11 - 98.0) and had marginally lower IQ estimates (coef = 0.69, t=2.98, p=0.03, 95%CI= 0.23 -1.14). Those with data on psychotic experiences at age 18 were of a similar age, had similar severity of negative and depression symptoms, but were more often female (X^2^=7.77, p=0.005) and had higher IQ scores (coef = 2.21; t=9.97; p< 0.001, 95%CI= 1.78- 2.64) than those without data available. Social functioning data were gathered at age 14, 16 and 18. Overall, 53.24% of the participants (n=1,116) completed all three social assessments, 29.10% (n=610) two, 17.32% (n=363) one assessment, and 0.33% (n=7) had no social functioning data available. Those without any, or with only one social functioning assessment did not differ from the rest of the sample in terms of age, gender distribution, positive, negative or depression symptoms, but had lower IQ estimates (coef = 1.90, t=6.64, p<0.001, 95%CI= 1.34- 2.47). The datasets generated during and/or analysed during the current study are available from the corresponding author on reasonable request.
