# Supplementary material for: Genetic risk for schizophrenia and autism, social impairment and developmental pathways to psychosis
Source: Transl Psychiatry. 2018 Sep 26;8:204. doi: 10.1038/s41398-018-0229-0 (PMC6158250; doi:10.1038/s41398-018-0229-0)
Supplement: Supplementary file 4 — Supplement 4. Different SEM models tested [file 41398_2018_229_MOESM4_ESM.docx]

Supplement 4. Different SEM models tested (standardized coefficients are displayed)

A. Fully connected model B. Without PRSscz&asd → IQ

C. Without PRSscz&asd 🡪 SF baseline D. Without PRSscz → SF follow-up

E. Without PRSscz&asd 🡪 fMRI social F. Without PRSasd → PEs

G. Without PEs → SF follow-up H. Without IQ 🡪 SF follow-up

I. Without sex 🡪 SF baseline& fMRIsocial J. Without IQ → SF baseline

K. Without IQ 🡪 PEs L. Without IQ 🡪 fMRI social

M. Without Sex → SF follow-up
