# Supplementary material for: Genetic risk for schizophrenia and autism, social impairment and developmental pathways to psychosis
Source: Transl Psychiatry. 2018 Sep 26;8:204. doi: 10.1038/s41398-018-0229-0 (PMC6158250; doi:10.1038/s41398-018-0229-0)
Supplement: Supplementary file 5 — Supplement 5. Sensitivity analyses [file 41398_2018_229_MOESM5_ESM.docx]

5. Sensitivity analyses

For sensitivity purposes, we repeated our SEM analyses using all available data (n=2,096), thus, including individuals with at least one missing data point. The model still had reasonably acceptable model fit (χ2(10) = 9.02, p=0.06; RMSEA = 0.024; 90%CI = 0.000- 0.046, SRMR=0.016; CFI=0.981), although the pathway leading from the ‘social brain’ network to PEs was no longer statistically significant (stand. coefficient= -0.05, p=0.637).

Supplementary figure 2. SEM model with estimated missing values
