# Supplementary material for: Genetic risk for schizophrenia and autism, social impairment and developmental pathways to psychosis
Source: Transl Psychiatry. 2018 Sep 26;8:204. doi: 10.1038/s41398-018-0229-0 (PMC6158250; doi:10.1038/s41398-018-0229-0)
Supplement: Supplementary file 6 — Supplement 6. Correlation matrix of variables included in the pathway models [file 41398_2018_229_MOESM6_ESM.docx]

**Supplement 6. Correlation matrix of variables included in the pathway models**

|  | **PRSasd** | **PRSscz** | **IQ** | **Sex** | **Center** | **SocfMRI** | **SocFunBL** | **SocFunFU** |
| --- | --- | --- | --- | --- | --- | --- | --- | --- |
| **PRSasd** |  |  |  |  |  |  |  |  |
| **PRSscz** | 0.120* |  |  |  |  |  |  |  |
| **IQ** | 0.000 | 0.000 |  |  |  |  |  |  |
| **Sex** | 0.000 | 0.000 | 0.066* |  |  |  |  |  |
| **Center** | 0.000 | 0.000 | 0.185* | 0.011 |  |  |  |  |
| **SocfMRI** | -0.003 | -0.021 | -0.044 | -0.017 | 0.024 |  |  |  |
| **SocFunBL** | 0.002 | 0.011 | -0.034 | -0.046* | -0.024 | 0.017 |  |  |
| **SocFunFU** | -0.063* | -0.015 | 0.001 | 0.054* | 0.007 | 0.011 | 0.276* |  |
| **PEs** | 0.089* | 0.081* | 0.071* | -0.163* | -0.049 | -0.028 | -0.124* | -0.318* |
